# Associations of clinicopathological factors with local treatment and survival outcome in elderly patients with ductal carcinoma *in situ*

**DOI:** 10.3389/fsurg.2023.1074980

**Published:** 2023-05-05

**Authors:** Xu Zhang, Yufei Zeng, Zheng Wang, Xiaosong Chen, Kunwei Shen

**Affiliations:** Department of General Surgery, Comprehensive Breast Health Center, Ruijin Hospital, Shanghai Jiaotong University School of Medicine, Shanghai, China

**Keywords:** breast cancer, ductal carcinoma *in situ*, elderly, surgery, radiotherapy

## Abstract

**Background:**

Local treatment for ductal carcinoma *in situ* (DCIS) remains controversial for elderly patients. This study aims to evaluate the association of local treatment, clinicopathological factors, and survival in elderly DCIS patients.

**Methods:**

Patients ≥ 60 years diagnosed with DCIS from January 2009 to December 2018 were retrospectively included. Local treatment including breast surgery, axillary lymph node (ALN) surgery, and radiotherapy were analyzed among subgroups (age of 60–69, 70–79, and ≥ 80 years), and their associations with clinicopathological features and prognostic outcome were further evaluated.

**Results:**

A total of 331 patients were included. Eventually 86 patients received breast conserving surgery (BCS) and 245 patients received mastectomy. ALN surgery was omitted in 62 patients. Age and tumor size were independent factors that influenced the breast and ALN surgery (*P *< 0.05). Compared with patients aging 60–69, patients ≥ 80 years were more likely to receive BCS (OR 4.28, 95% CI 1.33–13.78, *P *= 0.015) and be exempt from ALN surgery (OR 0.19, 95% CI 0.05–0.69, *P *= 0.011). Patients with tumor >1.5 cm were significantly less likely to receive BCS (OR 0.45, 95%CI 0.25–0.83, *P *= 0.011) and more likely to receive ALN surgery (OR 4.41, 95%CI 1.96–10.48, *P *= 0.001) compared to patients with tumor ≤ 1.5 cm. Postoperative radiotherapy was performed in 48.8% patients who received BCS. Age was the only factor that associated with the radiotherapy decision after BCS in elderly DCIS patients (*P *= 0.025). No significant recurrence-free survival difference was observed among patients receiving different local treatments.

**Conclusions:**

Age was related to the choice of local treatment in elderly DCIS patients, but different treatment patterns didn't impact disease outcome.

## Introduction

With the widespread application of screening mammography, more ductal carcinoma *in situ* (DCIS) has been detected over the past few decades. Currently, among all the newly-diagnosed breast cancer, one fifth was presented as DCIS (1, 2). Although DCIS was considered a rather indolent lesion itself, approximately 25% to 50% of them will progress into invasive ductal carcinoma (IDC) eventually. So far, the treatment backbone for DCIS is still surgery, in a similar manner as IDC tumor.

Elderly patients usually were presented with more comorbidities, and have relatively shorter life expectancies (3). Normally, a trend of treatment de-escalation exists among elderly breast cancer patients. Elderly patients with DCIS experience lower local recurrence rate than younger patients (4–6), therefore debate remains about how to select the optimal treatment for them. Some suggests that active surveillance may be safe for elderly patients with rather low risk DCIS, in order to avoid overtreatment and reduce morbidity caused by surgery. However, others argue that elderly patients had longer life expectancies now and should be treated with same standard as younger patients (7). Notably, elderly patients themselves are heterogeneous, with or without co-existing illness, and different kinds of illness all render them into different physical condition, resulting in different tolerance of local treatment. Currently, little is known regarding the clinical and pathological factors that contribute to treatment decisions in elderly DCIS patients.

Based on above issue, this study aims to evaluate the current local treatment patterns of elderly patients with DCIS. Also, we aim to explore the factors that influence the choice of local treatment and their associations with prognosis for elderly DCIS patients.

## Methods

### Study design and patients

Patients treated at the Comprehensive Breast Health Center, Ruijin Hospital from January 2009 and December 2018 were retrospectively reviewed. Elderly patients, defined as those aged ≥ 60 years, with a diagnosis of pure DCIS who received surgery with or without postoperative radiotherapy and had a minimum follow-up time of two years were included in this study. Main exclusion criteria included histologically proven invasive disease, metastatic breast cancer, and previously received treatment for DCIS. Demographic, diagnostic, clinicopathological, local treatment, follow-up and comorbidity information were retrieved from Shanghai Jiao Tong University Breast Cancer Database (SJTU-BCDB). Current study was performed in accordance with the Declaration of Helsinki and was approved by the institutional review board of Ruijin Hospital.

### Clinicopathological and follow-up data

All patients included received preoperative x-ray mammography and breast ultrasound evaluation. Full-field digital mammography with cranio-caudal and medio-lateral oblique views was applied and reviewed by experienced radiologists. Patients also underwent ultrasound examination of bilateral breast and axillary lymph nodes. A proportion of patients received breast MRI evaluation in a prone position on scanners having a field strength ≥1.5 T with a specified breast coil. Initial clinical manifestation at diagnosis were characterized as mass symptoms including palpable mass on physical examination or measurable mass on screening ultrasound, and non-mass symptoms including nipple discharge, or radiographic anomaly such as calcification or distortion on mammography. Patients enrolled received either mastectomy or breast conserving surgery (BCS) with definitive negative margin (>2 mm). Axillary lymph node (ALN) surgery, including sentinel lymph node biopsy (SLNB) and axillary lymph node dissection (ALND) was allowed. Expression of estrogen receptor (ER) and progesterone receptor (PR)were routinely detected by immunohistochemistry (IHC) in surgical specimens. The American Society of Clinical Oncology (ASCO) and the College of American Pathologists (CAP) guideline recommendations were used as criteria for categorizing ER and PR status (8). Nuclear grade was characterized into well differentiated (Grade I), intermediate (Grade II) or poorly differentiated lesions (Grade III). A recommendation of postoperative treatment including radiotherapy, endocrine therapy, or follow-up for each patient were made by a multidisciplinary consultation. Patients received BCS were considered postoperative radiotherapy. Patients with positive ER status who received BCS were routinely recommend endocrine therapy. For further subgroup analysis, patients were divided into different groups according to age: 60–69, 70–79, and ≥80 years.

Prognostic endpoints in this study included recurrence-free survival (RFS), defined as time from primary surgery to recurrence or metastasis of breast cancer, or death from any cause; and loco-regional recurrence (LRR), deﬁned as time from surgery to ipsilateral local or regional recurrence of either DCIS or invasive breast cancer. Last follow-up was completed by July 2021.

### Statistical analysis

All statistical analyses were performed using SPSS version 18.0 (SPSS, Inc., Chicago, IL). Statistical analyses included Chi-square test and multivariate logistic regression with odds ratio (OR) were used to assess the treatment recommendations in different patient groups. Time to recurrence was demonstrated by Kaplan–Meier curve and compared between groups using log-rank test. Subgroup analyses were performed by age (60–69, 70–79, ≥80 years old), breast surgery type (BCS or mastectomy), ALN surgery (yes or no), and radiotherapy (yes or no) following BCS. All statistical tests were two-tailed and statistical significance was defined as *P *< 0.05.

## Results

### Patient and clinicopathological characteristics

A total of 331 patients with complete clinicopathological and follow-up data were included in this study, with 242 (73.1%), 67 (20.3%), and 22 (6.6%) patients aged 60–69, 70–79, and ≥80 years, respectively. The mean age was 67.3 years (range, 60–90 years).

Patient and clinicopathological characteristics of the entire population were summarized in Table 1. A total of 60.4% patients presented with mass at diagnosis. According to pathology evaluation, 217 patients (65.6%) had tumors ≤ 1.5 cm, and 222 patients (67.1%) had ER-positive disease. In terms of biopsy method, 190 patients (57.4%) received core needle biopsy for diagnosis prior to surgery, and 141 (42.6%) patients received excisional biopsy prior or during surgery. Regarding comorbidity, 208 of 331 patients (62.8%) were accompanied with at least one co-existing disease (Supplementary Table S1).

**Table 1:** Patient and clinicopathological characteristics according to age subgroups.

Characteristics	Total, No. (%)	Age			*P*
		60–69, No. (%)	70–79, No. (%)	≥80, No. (%)	
Tumor size (cm)					0.358
≤1.5	217 (65.6%)	158 (65.3%)	41 (61.2%)	18 (81.8%)	
>1.5	114 (34.4)	84 (34.7%)	26 (38.8)	4 (18.2)	
Manifestation at diagnosis					0.246
Mass	200 (60.4%)	143 (59.1%)	40 (59.7%)	17 (77.3%)	
Non-mass	131 (39.6%)	99 (40.9%)	27 (40.3%)	5 (22.7%)	
Biopsy method					0.780
Core needle biopsy	190 (57.4%)	141 (58.3%)	36 (53.7%)	13 (59.1%)	
Excisional biopsy	141 (42.6%)	101 (41.7%)	31 (46.3%)	9 (40.9%)	
Nuclear grade	** **	** **	** **	** **	0.069
Low	78 (23.6%)	54 (22.3%)	18 (26.9%)	6 (27.3%)	
Intermediate	139 (42.0%)	93 (38.4%)	32 (47.7%)	14 (63.6%)	
High	108 (32.6%)	90 (37.2%)	16 (23.9%)	2 (9.1%)	
Unknown	6 (1.8%)	5 (2.1%)	1 (1.5%)	0 (0.0%)	
ER status	** **	** **	** **	** **	0.222
Positive	222 (67.1%)	155 (64.0%)	48 (71.6%)	19 (86.4%)	
Negative	101 (30.5%)	81 (33.5%)	17 (25.4%)	3 (13.6%)	
Unknown	8 (2.4%)	6 (2.5%)	2 (3.0%)	0 (0.0%)	
PR status					0.077
Positive	188 (56.8%)	128 (52.9%)	42 (62.7%)	18 (81.8%)	
Negative	135 (40.8%)	108 (44.6%)	23 (34.3%)	4 (18.2%)	
Unknown	8 (2.4%)	6 (2.5%)	2 (3.0%)	0 (0.0%)	
Number of comorbidities					**<0**.**001**
0	123 (37.2%)	105 (43.3%)	13 (19.4%)	5 (22.7%)	
1	112 (33.8%)	80 (33.1%)	26 (38.8%)	6 (27.3%)	
≥2	96 (29.0%)	57 (23.6%)	28 (41.8%)	11 (50.0%)	
CCI					**<0**.**001**
2	195 (58.9%)	195 (80.6%)	0 (0.0%)	0 (0.0%)	
3	93 (28.1%)	40 (16.5%)	38 (56.7%)	15 (68.2%)	
≥4	43 (13.0%)	7 (2.9%)	29 (43.3%)	7 (31.8%)	

ER, estrogen receptor; PR, progesterone receptor; CCI, Charlson Comorbidity Index.

Values of statistically significance, defined as *P*<0.05, were shown in bold.

Comparison of clinicopathological features among different age subgroups can also be found in Table 1. There was no significant difference in tumor size, manifestation at diagnosis, biopsy method, nuclear grade, ER status, and PR status among three age subgroups (all *P *> 0.05). While significantly more comorbidities (*P *< 0.001) and higher Charlson Comorbidity Index (CCI, *P *< 0.001) were observed in patients aged ≥80 years.

### Comparison of local treatment patterns among age groups

**Figure 1:**
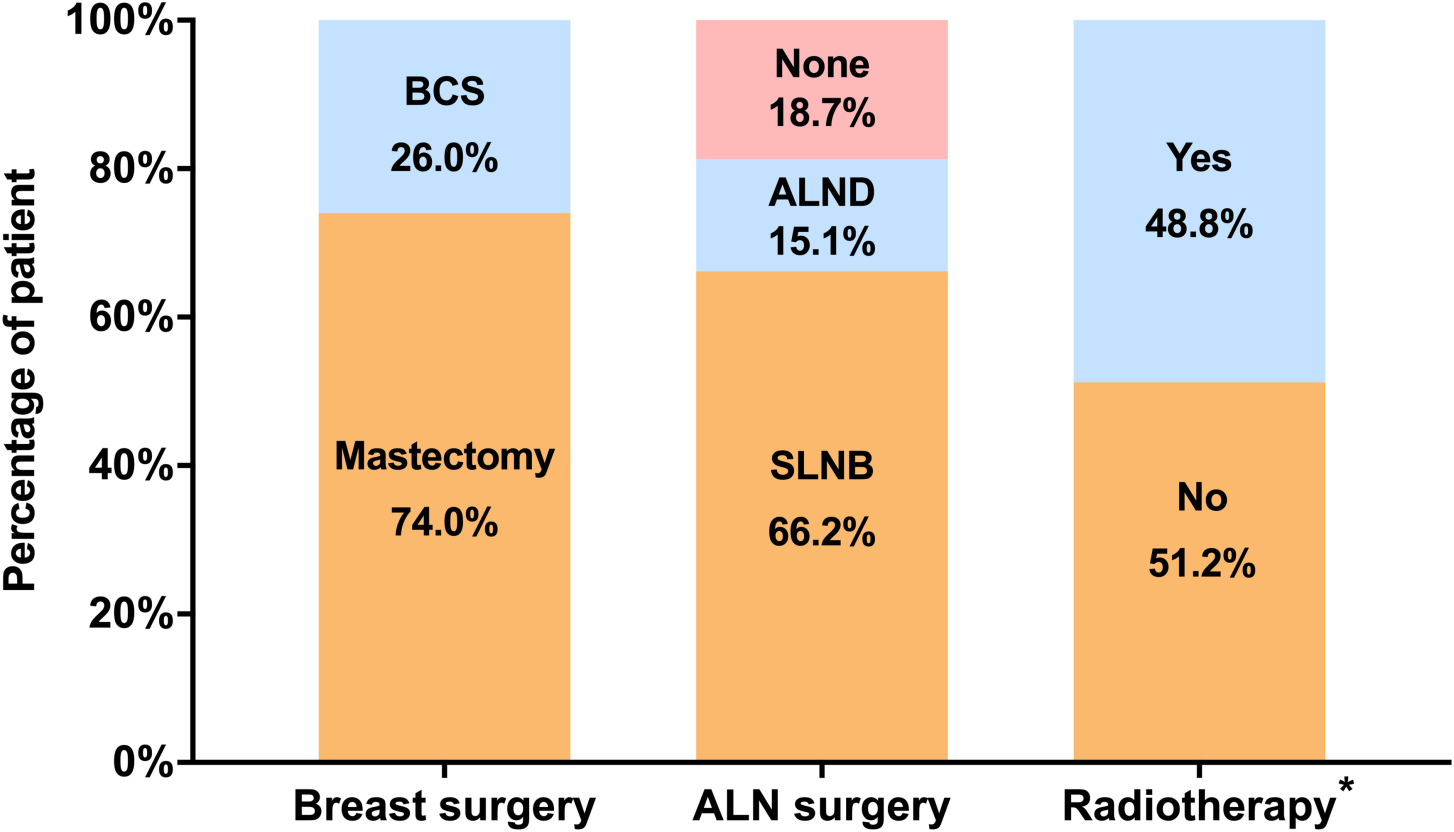
Distribution of local treatment patterns in elderly patients with DCIS. *Radiotherapy was considered in patients receiving BCS. (BCS, breast-conserving surgery; ALN, axillary lymph node; SLNB, sentinel lymph node biopsy, ALND, axillary lymph node dissection).

Local treatment patterns in elderly DCIS patients were listed in Figure 1. More patients received mastectomy (74.0%) rather than BCS (26.0%) as breast surgery. ALN surgery was performed in 269 (81.3%) patients, including 219 (66.2%) patients receiving SLNB and 50 (15.1%) receiving ALND. Among the 86 patients receiving BCS, only 42 (48.8%) patients were treated with postoperative radiation.

**Figure 2:**
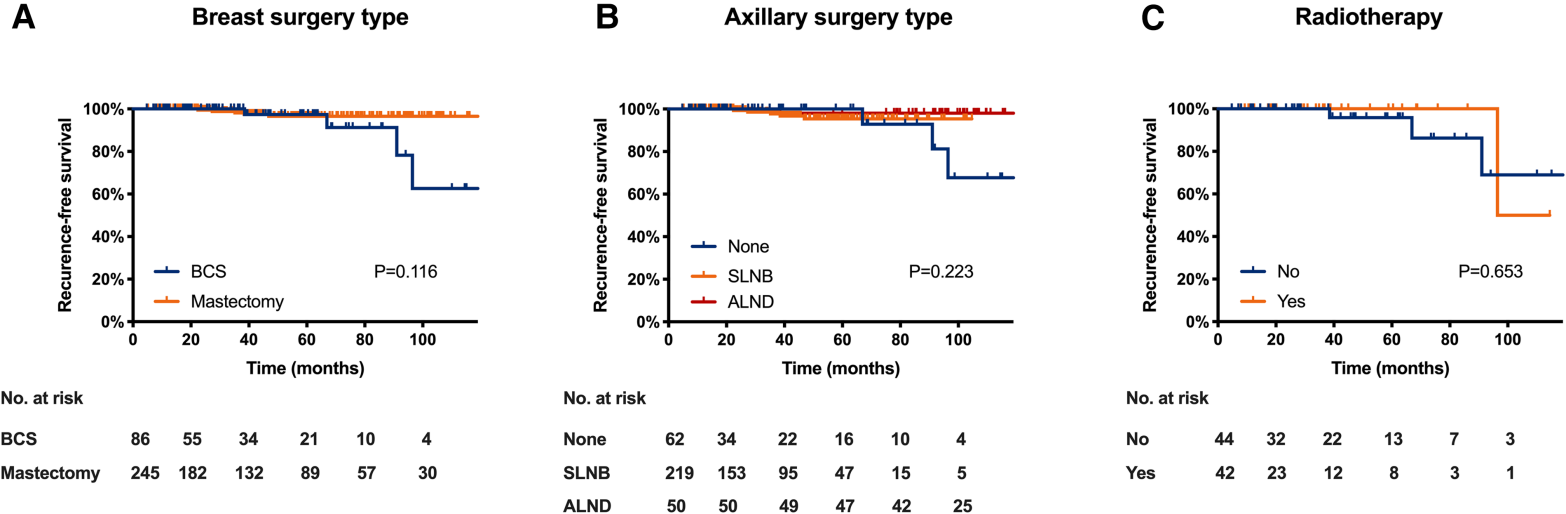
Distribution of local treatment methods by age subgroup. (**A**) distribution of breast surgery type; (**B**) distribution of axillary lymph node surgery; (**C**) distribution of radiotherapy in patients received BCS. (BCS, breast conserving surgery, SLNB, sentinel lymph node biopsy; ALND, axillary lymph node dissection).

Local treatment patterns were compared among three age subgroups (Figure 2). Patients ≥ 80 received significantly more BCS as breast surgery compared with those aged 60–69 (59.1% vs. 23.1%, *P *< 0.001) and 70–79 years (59.1% vs. 25.4%, *P *= 0.006). They also received less ALN surgery compared with patients aged 60–69 (50.0% vs. 16.1%, *P *< 0.001) and 70–79 years (50.0% vs. 17.9%, *P *= 0.010). For patients receiving BCS, omitting postoperative radiotherapy were more common in patients ≥ 80 compared with those aged 60–69 (84.6% vs. 42.9%, *P *= 0.007).

### Factors influencing breast surgery type

Age, tumor size, and manifestation at diagnosis were all significantly associated with the choice of breast surgery type according to univariate analysis (Supplementary Table S2). The proportion of patients receiving BCS were significantly different among 60–69, 70–79, and ≥ 80 age subgroups (*P *= 0.001). Patients with tumor size ≤ 1.5 cm received more BCS than those with tumor size >1.5 cm (30.4% vs. 17.5%, *P *= 0.011). In addition, the percentage of BCS was higher in patients presenting with mass at diagnosis than those with non-mass lesion (30.0% vs. 19.8%, *P *= 0.039).

Multivariate analysis demonstrated that age, tumor size, and manifestation at diagnosis remained to be independent factors for breast surgery type choice (Table 2). Compared with patients aged 60–69, those aged ≥ 80 were more likely to receive BCS [odds ratio (OR) 4.28, 95%CI 1.33–13.78; *P *= 0.015]. Patients with tumor > 1.5 cm were less likely to receive BCS compared with patients who had tumor ≤ 1.5 cm (OR 0.45, 95%CI 0.25–0.83; *P *= 0.011). Furthermore, BCS was more commonly performed in patients presenting with mass at diagnosis than those presenting with non-mass lesion (OR 1.96, 95%CI 1.11–3.45; *P *= 0.021). However, comorbidity and CCI had no significant effect on breast surgery choice for elderly patients with DCIS (both *P *> 0.05).

**Table 2:** Multivariate analysis of characteristics associated with different surgery types.

Variables	Receiving BCS^a^		Receiving ALN surgery^b^	
	OR (95% CI)	*P*	OR (95% CI)	*P*
Age	** **	**0**.**030**	** **	**0**.**016**
70–79 vs. 60–69 years	1.24 (0.46–3.35)	0.669	0.81 (0.25–2.63)	0.720
≥80 vs. 60–69 years	4.28 (1.33–13.78)	**0**.**015**	0.19 (0.05–0.69)	**0**.**011**
Tumor size >1.5 cm vs. ≤ 1.5 cm	0.45 (0.25–0.83)	**0**.**011**	4.41 (1.96–10.48)	**0**.**001**
Number of comorbidities		0.110		0.472
1 vs. 0	1.55 (0.82–2.95)	0.180	0.67 (0.32–1.38)	0.278
≥2 vs. 0	2.32 (1.05–5.15)	**0**.**038**	0.62 (0.25–1.55)	0.304
CCI		0.811		0.648
3 vs. 2	0.75 (0.31–1.80)	0.518	1.40 (0.49–4.01)	0.534
≥4 vs. 2	0.74 (0.19–2.80)	0.654	0.97 (0.20–4.64)	0.965
Mass vs. non-mass at diagnosis	1.96 (1.11–3.45)	**0**.**021**	0.89 (0.48–1.66)	0.708
Excisional biopsy vs. core needle biopsy	1.11 (0.65–1.91)	0.703	0.47 (0.25–0.87)	**0**.**017**

ALN, axillary lymph node.

CCI, Charlson comorbidity index.

^a^ As compared to receiving mastectomy.

^b^ As compared to receiving no ALN surgery.

Values of statistically significance, defined as *P*<0.05, were shown in bold.

### Factors influencing the choice of ALN surgery

Regarding axillary evaluation, age, tumor size, and breast biopsy type were all significantly related with the choice of ALN surgery in univariate analysis (Supplementary Table S2). Patients ≥ 80 were less likely to receive ALN surgery than those aged 60–69 and 70–79 (50% vs. 83.9% and 82.1%, *P *< 0.001). Patients with tumor >1.5 cm received more ALN surgery than patients with tumor ≤ 1.5 cm (93.9% vs. 74.7%, *P *< 0.001). In addition, ALN surgery was differently omitted in patients who received core needle biopsy and those directly received excisional biopsy (12.6% vs. 27.0%, *P *= 0.001).

Multivariate analysis revealed that age, tumor size, and breast biopsy type all remained to be independent predictors for performing ALN surgery (all *P *< 0.05, Table 2). Patients ≥ 80 were more often exempt from ALN surgery compared to those aged 60–69 (OR 0.19, 95%CI 0.05–0.69; *P *= 0.011). Patients with tumor >1.5 cm were more likely to receive ALN surgery than patients with tumor ≤ 1.5 cm (OR 4.41, 95%CI 1.96–10.48; *P *= 0.001). As for breast biopsy type, excisional biopsy led to a higher probability to omit ALN surgery compared with core needle biopsy (OR 0.47, 95%CI 0.25–0.87; *P *= 0.017).

### Factors influencing the decision of radiotherapy following BCS

Postoperative radiotherapy in elderly DCIS patients received BCS were commonly modified. Age was the only factor that significantly associated with the choice of radiotherapy following BCS (*P *= 0.025, Table 3). Compared with 60–69 age subgroup, patients ≥80 years were less likely to receive postoperative radiation (OR 0.14, 95%CI 0.27–0.67; *P *= 0.014). However, comorbidity and CCI were not associated with the decision of radiotherapy in elderly DCIS patients who received BCS (both *P *> 0.05).

**Table 3:** Clinicopathological characteristics associated with adjuvant radiotherapy in patients receiving breast conserving surgery.

Characteristics	Yes	No	*P*
	(*n* = 42)	(*n* = 44)	
Age (years)			0.025
60–69	32 (57.1%)	24 (42.9%)	
70–79	8 (47.1%)	9 (52.9%)	
≥80	2 (15.4%)	11 (84.6%)	
Tumor size (cm)			0.254
≤1.5	30 (45.5%)	36 (54.5%)	
>1.5	12 (60.0%)	8 (40.0%)	
Number of comorbidities			0.095
0	15 (62.5%)	9 (37.5%)	
1	16 (53.3%)	14 (46.7%)	
≥2	11 (34.4%)	21 (65.6%)	
CCI			0.139
2	26 (57.8%)	19 (42.2%)	
3	12 (44.4%)	15 (55.6%)	
≥4	4 (28.6%)	10 (71.4%)	
Manifestation at diagnosis			0.743
Mass	30 (50.0%)	30 (50.0%)	
No mass	12 (46.2%)	14 (53.8%)	
Nuclear grade			0.701
Low	12 (44.4%)	15 (55.6%)	
Intermediate	19 (41.3%)	27 (58.7%)	
High	11 (91.7%)	1 (8.3%)	
Unknown	0 (0.0%)	1 (100.0%)	
ER status			0.113
Positive	8 (66.7%)	4 (33.3%)	
Negative	32 (44.4%)	40 (55.6%)	
Unknown	2 (100.0%)	0 (0.0%)	
PR status			0.303
Positive	9 (52.9%)	8 (47.1%)	
Negative	31 (46.3%)	36 (53.7%)	
Unknown	2 (100.0%)	0 (0.0%)	

CCI, Charlson comorbidity index; ER, estrogen receptor; PR, progesterone receptor.

Values of statistically significance, defined as *P*<0.05, were shown in bold.

### Prognostic outcomes according to local treatment

In the study population, 72 of 331 (21.7%) patients underwent BCS had ER-positive disease. All these patients received standard endocrine treatment, among which 27 patients received aromatase inhibitor and 45 received tamoxifen. After a median follow-up of 52.2 months, 2 (0.6%) LRR events, 4 (1.2%) contralateral breast cancer, 1 (0.3%) distant metastasis, and 7 (2.1%) deaths were observed in the cohort (Supplementary Table S3). Among the 7 death events, 1 was breast cancer-related death, and 6 were death from other causes. RFS was statistically different among 60–69, 70–79, and ≥80 subgroups (*P *< 0.001, Supplementary Figure S1A). However, LRR was similar for patients aging 60–69, 70–79, and ≥80 (*P *= 0.698, Supplementary Figure S1B).

**Figure 3:**
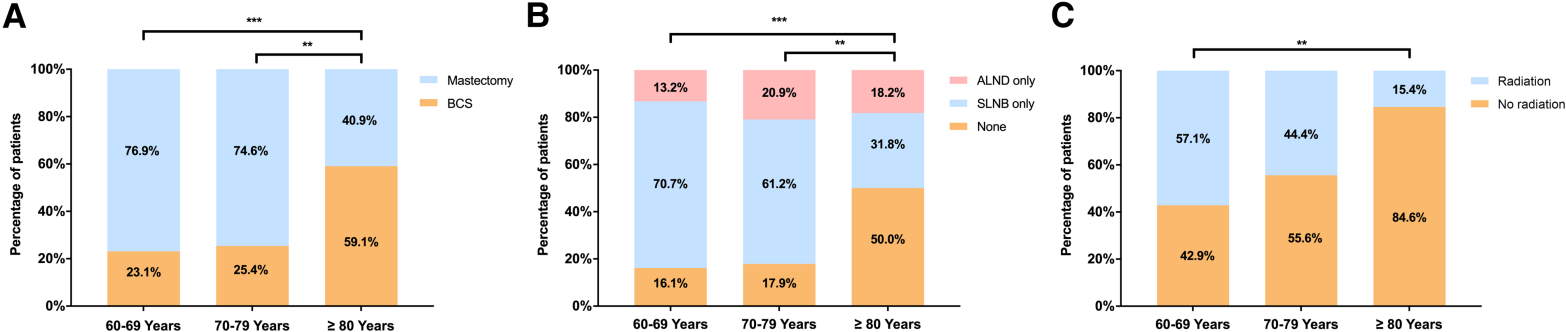
Recurrence-free survival in elderly patients with DCIS by (**A**) breast surgery; (**B**) ALN surgery; and (**C**) radiotherapy following BCS. (BCS, breast conserving surgery, SLNB, sentinel lymph node biopsy, ALND, axillary lymph node dissection).

Clinical outcomes were similar among patients receiving different local treatments (Figure 3). Comparable RFS was observed between patients receiving mastectomy and BCS (*P *= 0.146, Figure 3A). Similarly, patients receiving no ALN surgery, SLNB or ALND had comparable RFS (*P *= 0.363, Figure 3B). For patients underwent BCS, receiving radiotherapy or not have no significant impact on RFS (*P *= 0.468, Figure 3C).

## Discussion

The relatively indolent nature and good prognosis of DCIS raises concerns on its over-diagnosis and overtreatment, especially among elderly patients (9, 10). In this study, we found that age rather than comorbidity status significantly influence the choice of local treatments. Elderly DCIS patients appear to receive less aggressive surgery type and less adjuvant radiotherapy after BCS, but without impaired disease outcome.

Currently it's acknowledged that DCIS is a precursor lesion to most, if not all, invasive breast cancer (11). However, this progression is usually unpredictable and a considerable percentage of DCIS lesions will never become invasive (2). Compared with younger DCIS patients, the recurrence rate of DCIS in older women is lower. Considering limited life expectancy in elderly patients, less aggressive treatments are usually recommended (12, 13). However, the appropriate local treatment for elderly patients with DCIS is still controversial. Debate remains about the feasibility to choose active monitoring in substitution for surgery, to omit radiotherapy after BCS, or to spare axillary evaluation during mastectomy (14). Moreover, evidence is still scarce regarding factors influencing the choice of different local treatment.

Surgery is still regarded as the primary treatment for DCIS tumors. Although DCIS patients are eligible for either mastectomy or breast conserving surgery with equivalent safety and survival benefit (15), more than half of cases in our cohort underwent mastectomy, which is consistent with previous reports (16). Bleicher et al. found that older women with DCIS chose mastectomy over breast-conserving treatment if they have larger tumor size, lower education level, or consulted greater number of surgeons, while age and comorbidities did not predict choosing mastectomy (3). According to our results, patients older than 80 years were more likely to receive BCS than mastectomy. Moreover, patients with tumor size larger than 1.5 cm or primarily presented with mass symptom were less likely to receive BCS. This was not unexpected as for larger tumors, as BCS may be difficult to achieve clear resection margin. Among well-established risk factors for local recurrence in DCIS including histologic subtype, nuclear grade, and age, etc., margin status was described as the most important one (17, 18). Although tumor size was not identified as the predicting factor for LRR in DCIS according to NSABP B-17 or EORTC trials (19, 20), it is anticipated that larger tumor size and non-mass lesion might indicate an extensive lesion requiring mastectomy to ensure clear margin status. In this study, more than half (71%) patients received preoperative breast MRI evaluation, which might identify non-mass enhancement beyond target lesion found by mammography or ultrasound. Application of preoperative MRI were probably associated with decreased breast-conserving rate in this study. In addition, co-existing cardiovascular diseases would raise concern when considering radiotherapy after BCS. Patient's preference is an important factor that determine surgery type in China. Surgery type, BCS or mastectomy, was usually discussed by patients and her family members. Elderly patients care less about cosmetics but more about side effects and economics of radiotherapy. Therefore, mastectomy could be an option (21, 22).

As recommended, mastectomy is routinely accompanied by axillary evaluation for DCIS cases because subsequent sentinel lymph node biopsy would be difficult to perform if an invasive disease was found on postoperative pathological specimen (23). Especially in patients diagnosed with core needle biopsy since limited sample may lead to pathological underestimation (24). Consistently, in our study, compared with patients receiving excisional biopsy, patients receiving core needle biopsy prior to surgery were more likely to underwent axillary evaluation. We also observed that the percentage of patients receiving ALN surgery were significantly higher in mastectomy subgroup than BCS subgroup.

Less axillary evaluation was performed in elderly patients according to our results, especially for those older than 80 years. Furthermore, our study demonstrated that receiving ALN surgery or not have no impact on local recurrence. DCIS patients usually have no clinically detected lymph node. Although the final pathological diagnosis might be upgraded to invasive cancer, axillary lymph node metastasis and regional recurrence is still scarce for DCIS patients (19, 25). We admitted that the proportion of patients who received ALN surgery is relatively high. However, the real-world clinical practice in China is somewhat difficult to follow the treatment standard of DCIS, which did not recommend routine axillary evaluation for DCIS patients, especially for those received BCS. Chinese patients usually refuse a secondary ALN surgery if invasive disease is detected pathologically after primary surgery. Therefore, most patients demand ALN evaluation at the same time when they received breast surgery. Some radical patients request for a total mastectomy and axillary lymph node dissection even if no invasive breast cancer was found in preoperative biopsy. Though there exist the worries on a second surgery or locoregional recurrence from patients, the rate of upstaging from DCIS to invasive disease has been reported less than 20%. Most upstaged disease were Stage IA invasive ductal carcinoma, which have low risk of nodal metastasis (26). In recent years, we took great effort in patient education and found that the proportion of ALN surgery decreased in DCIS patients. Therefore, it is reasonable to presume ALN surgery could be omitted when performing BCS for elderly DCIS patients.

The benefit brought by postoperative radiotherapy in DCIS patients must be carefully weighed against the accompanying complications (27). With the development of modern radiation techniques, radiotherapy has been proved to be safe and has minimal impact on quality of life, and leading to limited cardiovascular mortality for the elderly (28–30). However, worries on deterioration of their comorbidities and inconvenient daily hospital visits for radiotherapy still trouble specialists and patients. According to our results, more than half of all patients were omitted of postoperative radiotherapy after BCS, and for patients older than 80 years, up to 84.6% were precluded with adjuvant radiotherapy. Age was the only factor related to radiotherapy after BCS. Consistently, Smith et al. had also observed that the proportion of receiving adjuvant radiotherapy decreased while patient age increased (31). Their study reported an omission of radiotherapy after BCS in 51.0% of all patients and 36.8%, 49.9% and 70.8% in patients aging 66–69, 70–79 and ≥80, respectively (*P* < 0.001).

A number of randomized clinical trials have already demonstrated that adding radiotherapy after surgery for DCIS patients of all ages could improve local control rate, which could reduce IBTR by approximately half (19, 32–36). However, the survival benefit brought by radiotherapy in elderly patients, especially patients over 80 years remained controversial. Smith et al. (31) found that radiotherapy after BCS contributed to a significant reduction in LRR in a group of DCIS patients over 65 years old. According to age subgroups, they found that healthy women of 66–79 years old were twice as likely to benefit from radiotherapy than patients ≥85 years who have moderate to severe comorbidity, leaving the benefit of radiotherapy for patients with rather old age no less debatable (31). Also, an EBCTCG meta-analysis had showed that radiotherapy resulted in a greater reduction in LRR for DCIS patients older than 50 years when compared with younger women, while no further study was conducted among patients with age over 65 or more (36).

On the contrary of supporting adjuvant radiotherapy for elderly patients, a study by Ho et al.(37) reported no LRR difference between patients receiving radiotherapy or not in women older than 60 years. Likewise, our study also found that elderly patients with different local treatment modalities (mastectomy, BCS plus radiotherapy, or BCS alone) shared similar LRR. Moreover, none of the patients ≥80 years in our study experienced LRR during follow-up. According to our inclusion criteria and clinicopathological characteristics, the high percentage of negative margin status and low Ki-67 index of enrolled patients probably reduced the potential benefit from radiotherapy (38). The role of adjuvant radiotherapy for elderly DCIS patients warrants further investigation since available evidence is limited.

DCIS is a group of disease with heterogeneous natural course and prognosis. A lot of effort has been made in risk stratification in order to identify a group of DCIS patients with good prognosis, in whom surgical excision alone or even observation could be enough to achieve a satisfactory local control. According to available prognostic factors for DCIS, older age could predict decreased risk of recurrence. Prognostic scores or multigene assays could also be used to evaluated the local control benefit offered by radiotherapy after BCS in DCIS patients (22, 39–42). In future perspective, local treatment strategies may be tailored according to recurrence stratification model in order to balance benefit and risk.

Our study has several limitations. Firstly, the number of patients included in our study was limited. And our follow-up time is relatively short, given the long natural history of DCIS. Therefore, the small number of outcome events may not provide sufficient statistical power to detect the benefit conferred by treatment. Secondly, this is a single institution retrospective study. Large-scaled prospective studies are warranted to validate our results.

In summary, our study presents the current approach of local treatment in elderly DCIS patients. Age is related with the choice of breast surgery, ALN surgery, and postoperative radiotherapy. DCIS patients with age ≥80 years old receive less aggressive local treatments but have no impaired disease outcome.

## Data Availability

The raw data supporting the conclusions of this article will be made available by the authors, without undue reservation.

## References

[1] Kuerer HM Albarracin CT Yang WT Cardiff RD Brewster AM Symmans WF Ductal carcinoma in situ: state of the science and roadmap to advance the field. J Clin Oncol. (2009) 27:279–88. 10.1200/JCO.2008.18.3103 19064970

[2] Ward EM DeSantis CE Lin CC Kramer JL Jemal A Kohler B Cancer statistics: breast cancer in situ. CA Cancer J Clin. (2015) 65:481–95. 10.3322/caac.21321 26431342

[3] Bleicher RJ Abrahamse P Hawley ST Katz SJ Morrow M. The influence of age on the breast surgery decision-making process. Ann Surg Oncol. (2008) 15:854–62. 10.1245/s10434-007-9708-x 18058182

[4] Vicini FA Recht A. Age at diagnosis and outcome for women with ductal carcinoma-in-situ of the breast: a critical review of the literature. J Clin Oncol. (2002) 20:2736–44. 10.1200/JCO.2002.07.137 12039936

[5] Kerlikowske K Molinaro A Cha I Ljung BM Ernster VL Stewart K Characteristics associated with recurrence among women with ductal carcinoma in situ treated by lumpectomy. J Natl Cancer Inst. (2003) 95:1692–702. 10.1093/jnci/djg097 14625260

[6] Solin LJ Fourquet A Vicini FA Taylor M Olivotto IA Haffty B Long-term outcome after breast-conservation treatment with radiation for mammographically detected ductal carcinoma in situ of the breast. Cancer. (2005) 103:1137–46. 10.1002/cncr.20886 15674853

[7] Merrill AL Esserman L Morrow M. Clinical decisions. Ductal carcinoma in situ. N Engl J Med. (2016) 374:390–2. 10.1056/NEJMclde1512213 26816018

[8] Allison KH Hammond MEH Dowsett M McKernin SE Carey LA Fitzgibbons PL Estrogen and progesterone receptor testing in breast cancer: american society of clinical oncology/college of American pathologists guideline update. Arch Pathol Lab Med. (2020) 144:545–63. 10.5858/arpa.2019-0904-SA 31928354

[9] Esserman LJ Thompson IM Jr. Reid B. Overdiagnosis and overtreatment in cancer: an opportunity for improvement. JAMA. (2013) 310:797–8. 10.1001/jama.2013.108415 23896967

[10] Benson JR Jatoi I Toi M. Treatment of low-risk ductal carcinoma in situ: is nothing better than something? Lancet Oncol. (2016) 17:e442–e51. 10.1016/S1470-2045(16)30367-9 27733270

[11] Espina V Liotta LA. What is the malignant nature of human ductal carcinoma in situ? Nat Rev Cancer. (2011) 11:68–75. 10.1038/nrc2950 21150936 PMC3756606

[12] Gennari R Curigliano G Rotmensz N Robertson C Colleoni M Zurrida S Breast carcinoma in elderly women: features of disease presentation, choice of local and systemic treatments compared with younger postmenopasual patients. Cancer. (2004) 101:1302–10. 10.1002/cncr.20535 15316944

[13] Muss HB. Adjuvant chemotherapy in older women with breast cancer: who and what? J Clin Oncol. (2014) 32:1996–2000. 10.1200/JCO.2013.54.8586 24868030

[14] van Seijen M Lips EH Thompson AM Nik-Zainal S Futreal A Hwang ES Ductal carcinoma in situ: to treat or not to treat, that is the question. Br J Cancer. (2019) 121:285–92. 10.1038/s41416-019-0478-6 31285590 PMC6697179

[15] Clarke M Collins R Darby S Davies C Elphinstone P Evans V Effects of radiotherapy and of differences in the extent of surgery for early breast cancer on local recurrence and 15-year survival: an overview of the randomised trials. Lancet. (2005) 366:2087–106. 10.1016/S0140-6736(05)67887-7 16360786

[16] Wyld L Garg DK Kumar ID Brown H Reed MW. Stage and treatment variation with age in postmenopausal women with breast cancer: compliance with guidelines. Br J Cancer. (2004) 90:1486–91. 10.1038/sj.bjc.6601742 15083173 PMC2409727

[17] Bijker N Peterse JL Duchateau L Julien JP Fentiman IS Duval C Risk factors for recurrence and metastasis after breast-conserving therapy for ductal carcinoma-in-situ: analysis of European organization for research and treatment of cancer trial 10853. J Clin Oncol. (2001) 19:2263–71. 10.1200/JCO.2001.19.8.2263 11304780

[18] Silverstein MJ Lagios MD Groshen S Waisman JR Lewinsky BS Martino S The influence of margin width on local control of ductal carcinoma in situ of the breast. N Engl J Med. (1999) 340:1455–61. 10.1056/NEJM199905133401902 10320383

[19] Fisher B Dignam J Wolmark N Mamounas E Costantino J Poller W Lumpectomy and radiation therapy for the treatment of intraductal breast cancer: findings from national surgical adjuvant breast and bowel project B-17. J Clin Oncol. (1998) 16:441–52. 10.1200/JCO.1998.16.2.441 9469327

[20] Julien JP Bijker N Fentiman IS Peterse JL Delledonne V Rouanet P Radiotherapy in breast-conserving treatment for ductal carcinoma in situ: first results of the EORTC randomised phase III trial 10853. EORTC breast cancer cooperative group and EORTC radiotherapy group. Lancet. (2000) 355:528–33. 10.1016/S0140-6736(99)06341-2 10683002

[21] Hughes LL Wang M Page DL Gray R Solin LJ Davidson NE Local excision alone without irradiation for ductal carcinoma in situ of the breast: a trial of the eastern cooperative oncology group. J Clin Oncol. (2009) 27:5319–24. 10.1200/JCO.2009.21.8560 19826126 PMC2773217

[22] Solin LJ Gray R Baehner FL Butler SM Hughes LL Yoshizawa C A multigene expression assay to predict local recurrence risk for ductal carcinoma in situ of the breast. J Natl Cancer Inst. (2013) 105:701–10. 10.1093/jnci/djt067 23641039 PMC3653823

[23] Tunon-de-Lara C Chauvet MP Baranzelli MC Baron M Piquenot J Le-Bouédec G The role of sentinel lymph node biopsy and factors associated with invasion in extensive DCIS of the breast treated by mastectomy: the cinnamome prospective multicenter study. Ann Surg Oncol. (2015) 22:3853–60. 10.1245/s10434-015-4476-5 25777085 PMC4595535

[24] Pilewskie M Karsten M Radosa J Eaton A King TA. Is sentinel lymph node biopsy indicated at completion mastectomy for ductal carcinoma in situ? Ann Surg Oncol. (2016) 23:2229–34. 10.1245/s10434-016-5145-z 26960927 PMC4943570

[25] Allred DC Anderson SJ Paik S Wickerham DL Nagtegaal ID Swain SM Adjuvant tamoxifen reduces subsequent breast cancer in women with estrogen receptor-positive ductal carcinoma in situ: a study based on NSABP protocol B-24. J Clin Oncol. (2012) 30:1268–73. 10.1200/JCO.2010.34.0141 22393101 PMC3341142

[26] Lars JG Marc DR Ann HP Alastair MT Jeremy ST Jelle W Surgical upstaging rates for vacuum assisted biopsy proven DCIS: implications for active surveillance trials. Ann Surg Oncol. (2017) 24:3534–40. 10.1245/s10434-017-6018-9 28795370 PMC6414216

[27] Barrio AV Van Zee KJ. Controversies in the treatment of ductal carcinoma in situ. Annu Rev Med. (2017) 68:197–211. 10.1146/annurev-med-050715-104920 28099081 PMC5532880

[28] Rayan G Dawson LA Bezjak A Lau A Fyles AW Yi QL Prospective comparison of breast pain in patients participating in a randomized trial of breast-conserving surgery and tamoxifen with or without radiotherapy. Int J Radiat Oncol Biol Phys. (2003) 55:154–61. 10.1016/S0360-3016(02)03826-9 12504048

[29] Darby SC Ewertz M McGale P Bennet AM Blom-Goldman U Bronnum D Risk of ischemic heart disease in women after radiotherapy for breast cancer. N Engl J Med. (2013) 368:987–98. 10.1056/NEJMoa1209825 23484825

[30] Darby S McGale P Peto R Granath F Hall P Ekbom A. Mortality from cardiovascular disease more than 10 years after radiotherapy for breast cancer: nationwide cohort study of 90 000 Swedish women. Br Med J. (2003) 326:256–7. 10.1136/bmj.326.7383.256 12560277 PMC140764

[31] Smith BD Haffty BG Buchholz TA Smith GL Galusha DH Bekelman JE Effectiveness of radiation therapy in older women with ductal carcinoma in situ. J Natl Cancer Inst. (2006) 98:1302–10. 10.1093/jnci/djj359 16985249

[32] Wapnir IL Dignam JJ Fisher B Mamounas EP Anderson SJ Julian TB Long-term outcomes of invasive ipsilateral breast tumor recurrences after lumpectomy in NSABP B-17 and B-24 randomized clinical trials for DCIS. J Natl Cancer Inst. (2011) 103:478–88. 10.1093/jnci/djr027 21398619 PMC3107729

[33] Donker M Litiere S Werutsky G Julien JP Fentiman IS Agresti R Breast-conserving treatment with or without radiotherapy in ductal carcinoma in situ: 15-year recurrence rates and outcome after a recurrence, from the EORTC 10853 randomized phase III trial. J Clin Oncol. (2013) 31:4054–9. 10.1200/JCO.2013.49.5077 24043739

[34] Warnberg F Garmo H Emdin S Hedberg V Adwall L Sandelin K Effect of radiotherapy after breast-conserving surgery for ductal carcinoma in situ: 20 years follow-up in the randomized SweDCIS trial. J Clin Oncol. (2014) 32:3613–8. 10.1200/JCO.2014.56.2595 25311220

[35] McCormick B Winter K Hudis C Kuerer HM Rakovitch E Smith BL RTOG 9804: a prospective randomized trial for good-risk ductal carcinoma in situ comparing radiotherapy with observation. J Clin Oncol. (2015) 33:709–15. 10.1200/JCO.2014.57.9029 25605856 PMC4334775

[36] Early Breast Cancer Trialists’ Collaborative G, Correa C McGale P Taylor C Wang Y Clarke M Overview of the randomized trials of radiotherapy in ductal carcinoma in situ of the breast. J Natl Cancer Inst Monogr. 2010;2010:162–77. 10.1093/jncimonographs/llgq039 20956824 PMC5161078

[37] Ho A Goenka A Ishill N Van Zee K McLane A Gonzales AM The effect of age in the outcome and treatment of older women with ductal carcinoma in situ. Breast. (2011) 20:71–7. 10.1016/j.breast.2010.07.005 20739181

[38] Lazzeroni M Guerrieri-Gonzaga A Botteri E Leonardi MC Rotmensz N Serrano D Tailoring treatment for ductal intraepithelial neoplasia of the breast according to Ki-67 and molecular phenotype. Br J Cancer. (2013) 108:1593–601. 10.1038/bjc.2013.147 23579208 PMC3668474

[39] Silverstein MJ Lagios MD. Choosing treatment for patients with ductal carcinoma in situ: fine tuning the university of southern California/van nuys prognostic Index. J Natl Cancer Inst Monogr. (2010) 2010:193–6. 10.1093/jncimonographs/lgq040 20956828 PMC5161065

[40] Rudloff U Jacks LM Goldberg JI Wynveen CA Brogi E Patil S Nomogram for predicting the risk of local recurrence after breast-conserving surgery for ductal carcinoma in situ. J Clin Oncol. (2010) 28:3762–9. 10.1200/JCO.2009.26.8847 20625132

[41] Sagara Y Freedman RA Vaz-Luis I Mallory MA Wong SM Aydogan F Patient prognostic score and associations with survival improvement offered by radiotherapy after breast-conserving surgery for ductal carcinoma in situ: a population-based longitudinal cohort study. J Clin Oncol. (2016) 34:1190–6. 10.1200/JCO.2015.65.1869 26834064 PMC4872326

[42] Rakovitch E Nofech-Mozes S Hanna W Baehner FL Saskin R Butler SM A population-based validation study of the DCIS score predicting recurrence risk in individuals treated by breast-conserving surgery alone. Breast Cancer Res Treat. (2015) 152:389–98. 10.1007/s10549-015-3464-6 26119102 PMC4491104

