# Supplementary material for: Associations of clinicopathological factors with local treatment and survival outcome in elderly patients with ductal carcinoma in situ
Source: Front Surg. 2023 May 5;10:1074980. doi: 10.3389/fsurg.2023.1074980 (PMC10196260; doi:10.3389/fsurg.2023.1074980)
Supplement: Supplementary file 1 [file Table1.docx]

**Table S1.** Comorbidity details of patients enrolled

| **Comorbidity** | **Number** |
| --- | --- |
| Hypertension | 163 |
| Diabetes | 39 |
| Hypo-/hyperthyroidism | 22 |
| Arrhythmia | 19 |
| Coronary heart disease | 13 |
| CVA or TIA | 8 |
| Mild pulmonary disease* | 8 |
| Mild liver disease** | 6 |
| Psychological illnesses | 5 |
| Mild renal disease*** | 5 |
| Connective tissue disease | 4 |
| Aortic dissection | 1 |
| Moderate liver disease**** | 1 |
| Peptic ulcer disease | 1 |
| Parkinson | 1 |
| Rheumatic heart disease | 1 |
| Ulcerative Colitis | 1 |

*Mild pulmonary disease = pulmonary disease that did not meet the criteria of Charlson Comorbidity Index for “chronic pulmonary disease”; ** Mild liver disease = Patients with chronic hepatitis or cirrhosis without portal hypertension; *** Mild renal disease = renal disease that did not meet the criteria of Charlson Comorbidity Index for “moderate CKD (creatinine > 270μmol/L)”; **** Moderate liver disease = Patients with cirrhosis with portal hypertension without a history of variceal bleeding.

Abbreviation: CVA = cerebrovascular accident; TIA = transient ischemic attack.

**Table S2.** Patient and clinicopathological characteristics in patients receiving different surgery types

| Characteristics | Breast surgery type | | |  | Receiving ALN surgery | | |
| --- | --- | --- | --- | --- | --- | --- | --- |
|  | **BCS** | **Mastectomy** | ***P*** |  | **Yes** | **No** | ***P*** |
|  | **(n=86)** | **(n=245)** |  |  | **(n=269)** | **(n=62)** |  |
| Age (years) |  |  | **0.001** |  |  |  | **<0.001** |
| 60-69 | 56 (23.1%) | 186 (76.9%) |  |  | 203 (83.9%) | 39 (16.1%) |  |
| 70-79 | 17 (25.4%) | 50 (74.6%) |  |  | 55 (82.1%) | 12 (17.9%) |  |
| ≥ 80 | 13 (59.1%) | 9 (40.9%) |  |  | 11 (50.0%) | 11 (50.0%) |  |
| Tumor size (cm) |  |  | **0.011** |  |  |  | **<0.001** |
| ≤ 1.5 | 66 (30.4%) | 151 (69.6%) |  |  | 162 (74.7%) | 55 (25.3%) |  |
| >1.5 | 20 (17.5%) | 94 (82.5%) |  |  | 107 (93.9%) | 7 (6.1%) |  |
| Number of comorbidities |  |  | 0.067 |  |  |  | 0.141 |
| 0 | 24 (19.5%) | 99 (80.5%) |  |  | 105 (85.4%) | 32 (14.6%) |  |
| 1 | 30 (26.8%) | 82 (73.2%) |  |  | 90 (80.4%) | 19 (19.6%) |  |
| ≥ 2 | 32 (33.3%) | 64 (66.7%) |  |  | 74 (77.1%) | 11 (22.9%) |  |
| CCI |  |  | 0.321 |  |  |  | 0.334 |
| 2 | 45 (23.1%) | 150 (76.9%) |  |  | 163 (83.6%) | 32 (16.4%) |  |
| 3 | 27 (29.0%) | 66 (71.0%) |  |  | 74 (79.6%) | 19 (20.4%) |  |
| ≥ 4 | 14 (32.6%) | 29 (67.4%) |  |  | 32 (74.4%) | 11 (25.6%) |  |
| Manifestation at diagnosis |  |  | **0.039** |  |  |  | 0.674 |
| Mass | 60 (30.0%) | 140 (70.0%) |  |  | 164 (82.0%) | 36 (18.0%) |  |
| Non-mass | 26 (19.8%) | 105 (80.2%) |  |  | 105 (80.2%) | 26 (19.8%) |  |
| Biopsy type |  |  | 0.549 |  |  |  | **0.001** |
| Core needle biopsy | 47 (24.7%) | 143 (75.3%) |  |  | 166 (87.4%) | 24 (12.6%) |  |
| Excisional biopsy | 39 (27.7%) | 102 (72.3%) |  |  | 103 (73.0%) | 38 (27.0%) |  |

Abbreviation: ALN, axillary lymph node; CCI, Charlson comorbidity index.

**Table S3.** Summary of recurrence and death events by age subgroup

| Age group | LRR | Contralateral breast cancer | Distant metastasis | Death | |
| --- | --- | --- | --- | --- | --- |
|  |  |  |  | **breast cancer** | **other causes** |
| Overall | 2 | 4 | 1 | 1 | 6 |
| 60-69 years | 1 | 2 | 0 | 0 | 1 |
| 70-79 years | 1 | 2 | 1 | 0 | 1 |
| ≥80 years | 0 | 0 | 0 | 1 | 4 |

Abbreviation: LRR = locoregional recurrence.


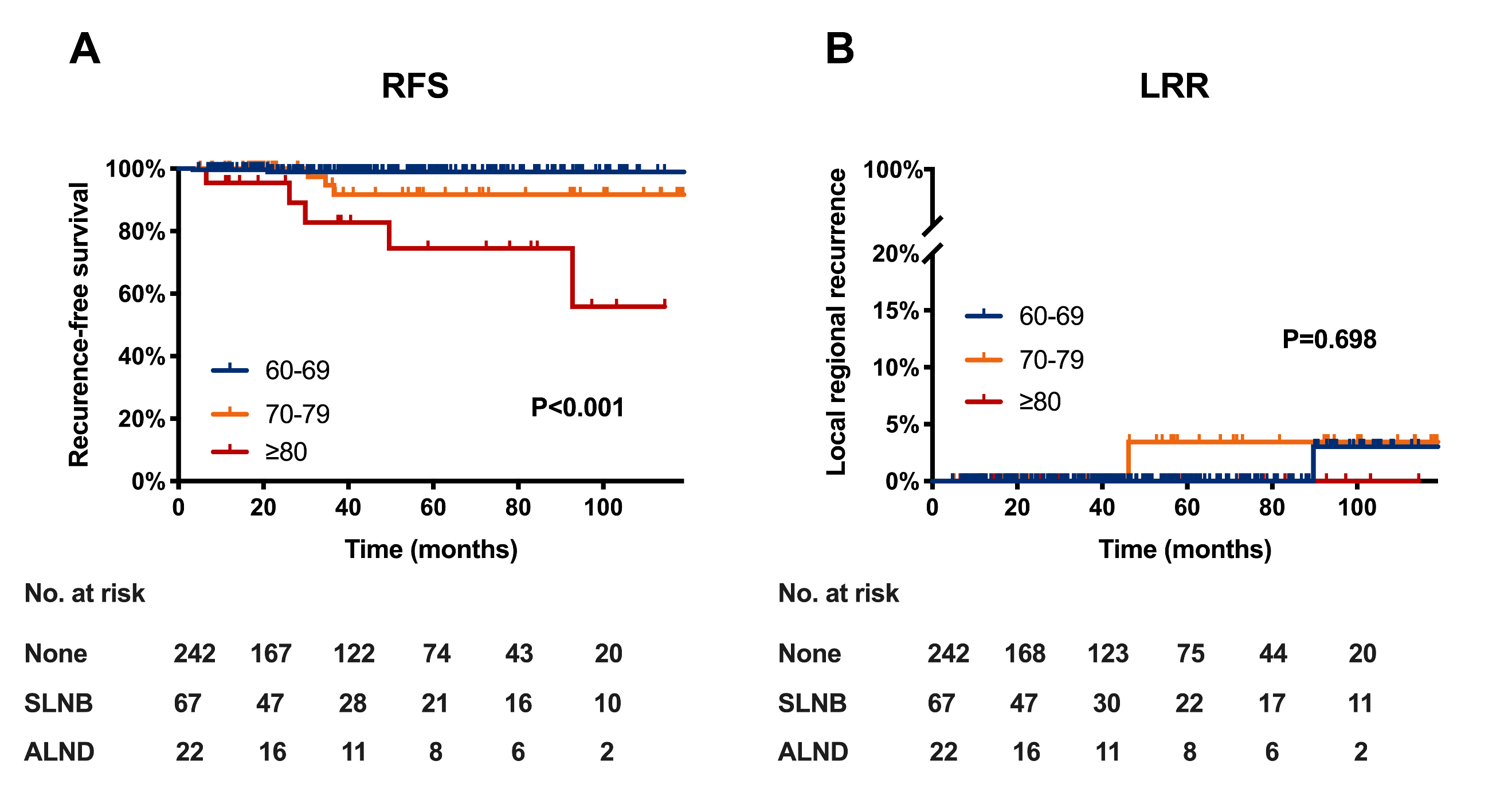


**Figure S1.** (**A**) Recurrence-free survival and (**B**) loco-regional recurrence (LRR) in elderly patients by age groups.
