# Supplementary figures and images for: Associations of clinicopathological factors with local treatment and survival outcome in elderly patients with ductal carcinoma in situ
Source: Front Surg. 2023 May 5;10:1074980. doi: 10.3389/fsurg.2023.1074980 (PMC10196260; doi:10.3389/fsurg.2023.1074980)

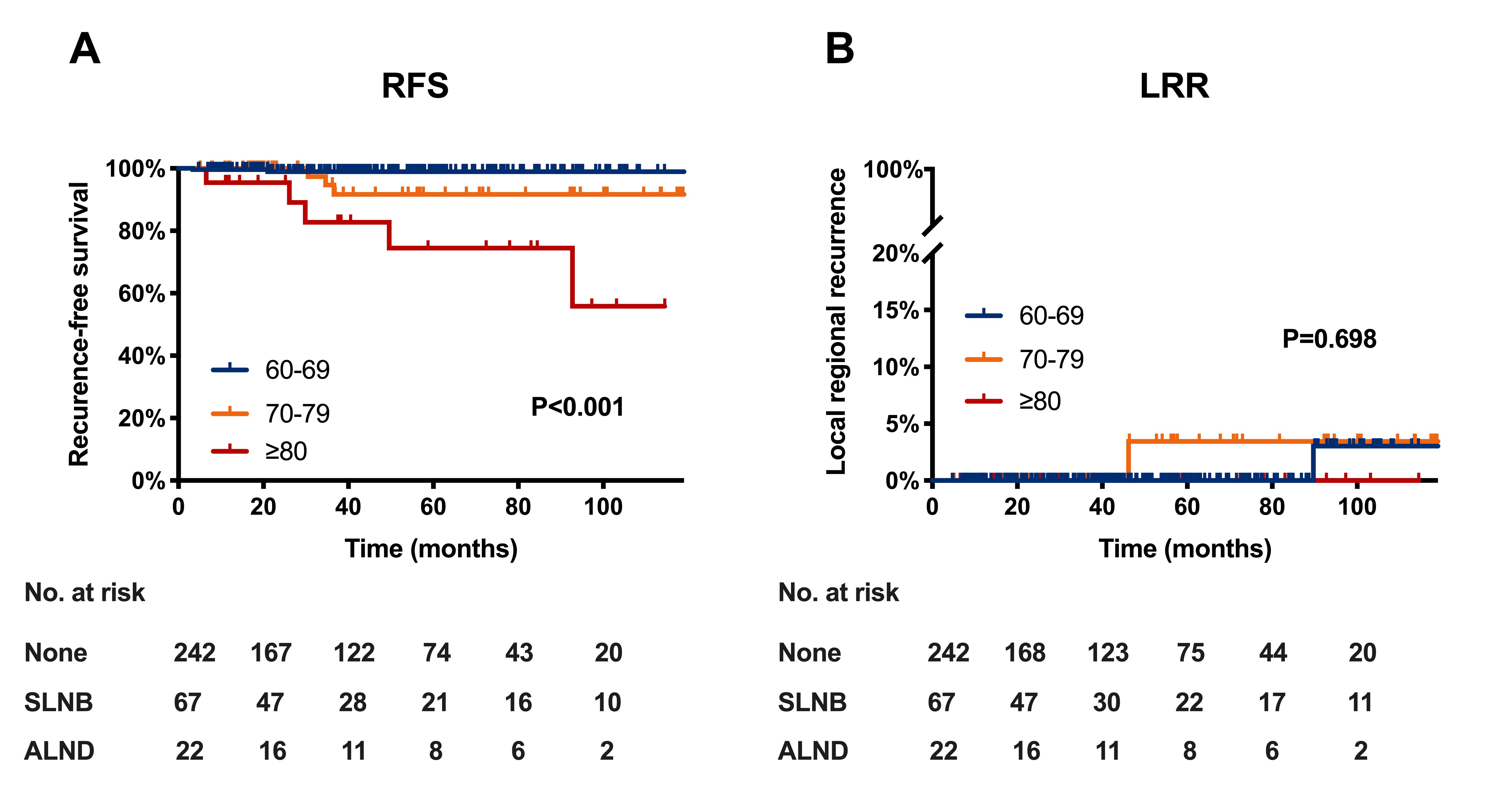

Supplement: Supplementary file 2 [file Image1.tiff]
